# Abstract of Remarks on Diseases of the Ear and Their Treatment

**Published:** 1871-10

**Authors:** Charles E. Rider


ART. II.—*Abstract of remarks on Diseases of the Ear and their Treatment.* By DR. CHARLES E. RIDER, before the Rochester City Medical Society, September 4th, 1871.

At the present time there exists considerable activity in Otology. In Germany two journals devoted to this subject are now published, and there exist in the German language several complete treatises on Aural Surgery. In our own country, Dr. Knapp of New York edits a journal of Ophthalmology and Otology, and we have an American Otological Society, at whose annual meeting many interesting facts and theories are advanced. The published proceedings of this Society furnish to the profession a condensed summary of the advances which have been made during the year in this department. We also have a few men, as for instance, Dr. J. Orne Green of Boston, who devote their entire time to the investigation of diseases of the organs of hearing.

We may for convenience study the disorders of hearing as they affect the external, middle, or internal ear.

1st. Diseases of the External Ear—Eczematous Inflammation. We meet this affection quite frequently, especially among badly nourished children, often existing just back of the auricle.

Locally, I apply the following Ointment:

℞ Yellow oxide of Mercury, - - - gr. xx.
Simple Cerate, - - - - ℥j
M.

Internally, I give good nourishment and tonics. Where the Eczema is rather dry, the best remedy perhaps is Fowler's Solution of Arsenic,—drops x, 3 times a day; but where the Eczema is more moist, I have used the following formula with much benefit:—

℞ Nitric Acid, - - - - - - ʒj.

Epsom Salts, - - - - ℥j.

Water, - - - - - - Oj.

M.

Give a tablespoonful three times a day.

Otitis Externa (diffuse otitis), commonly shows itself by an exfoliation of cuticle, with a slight purulent discharge. Sometimes, however, there may exist merely redness and dryness of the membrane, without any discharge; or even the disease may show an absence only of the normal amount of cerumen. For this trouble, we must correct any constitutional vice. Astringents are indicated for local use.

Furuncular Inflammation.—This affection is extremely painful and persistent, and often very difficult to cure. As soon as one begins to push up toward the centre of the meatus, immediately puncture it with a sharp, narrow bistoury; this generally relieves the pain; hot fomentations may follow. Dr. Von Troeltsch recommends the use of arsenic in this disease.

Dr. W. W. Ely has failed to obtain benefit in these cases by freely administering quinine and producing cinchonism; but where the severity and number of these furuncles are excessive, he has seen the following treatment invariably break up their course:—

℞ Hydrargyri Chloridi Corrosivi, gr. j. }

Fl. Ext. Sennæ et Jalapae, ℥j mx. } s. ʒj. ter die.

Accumulation of Inspissated Cerumen.—Against this we have no prophylactic, and if removed, it will usually re-accumulate at intervals of a year or more. With a syringe, as for instance, Mattison's, *gently* throw into the meatus a continuous stream of warm water; this will generally soften and wash out the mass. Always, ere any treatment is used, be sure that there is an abnormal amount of wax present; as injections of even warm water in a healthy ear may cause injury. Never use any instruments to remove the cerumen, as the membrana tympani is very easily injured.

Foreign Bodies in the External Meatus.—Always first examine carefully, to ascertain if such foreign body is in the meatus. In nine cases out of ten we shall find nothing present, as it is very difficult to introduce a foreign body into the meatus. Then remove with the syringe and warm water if possible, if not, use any suitable instrument, but only under the eye. Never introduce any instrument unless the eye sees its every motion.

Ear Ache.—Make warm water applications, or hot chamomile fomentations.

2d.—The Membrana Tympani is seldom diseased alone; but i often involved in inflammations occurring in the external, or middle ear.

3d. Diseases of the Middle Ear.—The cavity of the middle ear is lined by mucous membrane, which is a continuation through the eustachian tubes of that of the pharynx. Inflammation of this membrane is by far the most common disease of the ear.

Acute Otitis Media.—Almost every one, at some time, suffers from one or more slight attacks of otitis media. It is in fact a "cold" in the ear. We have slight pain, redness of membrana tympani, slight deafness, sensation as of water in the ear. Therapeutically we treat it as we would a common cold; keep quiet and apply to neck and feet derivative remedies.

Chronic Otitis Media.—This comes on very slowly, increasing very gradually; may result from repeated attacks of acute inflammation, but more often is slow and chronic from its inception. In In this complaint quite frequently the patient does not notice any trouble until the membrana by repairing is much thickened, and the hearing considerably impaired. If the hearing progressively grows worse, and if it has no remissions or periodical seasons of comparative improvement, we have but little hope from any treatment, as any and all therapeutical measures seem to fail in improving hearing.

Subacute Inflammation.—We may, however, speak of what we would call Subacute Otitis Media. In this form we have repeated attacks of acute inflammation, each leaving the ear worse than it found it, and although perhaps to a skilled observer the membrana tympani may appear somewhat less thickened than in the last described disease, still the diagnostic distinction is found in the fact that the hearing varies, being sensibly better at some times than at others, and in this variety treatment is often very beneficial, perhaps even curative. Here if we find coexisting naso-pharyngeal catarrh the use of the atomizer is indicated. Probably the best remedy to use therewith is a saturated solution of Chlorate of Potassa. Also we frequently are obliged to use the Eustachian Catheter. (I much prefer the hard rubber Catheter, to which by heat any desired curve can be given). Its use is not a painful, but is often an exceedingly delicate operation; as we so frequently meet with Septa nasi, which are so bent that it is very difficult, almost impossible, to introduce it successfully. Also in conjunction with the Catheter, or Politzer apparatus, we often find advantage in throwing up medicated vapours; as that of Tr. of Iodine or Tr. of Iron; and even in blowing up finely powdered Alum or Iodoform. I consider it however more than dangerous to try to throw up solutions into the middle ear

Purulent Otitis Media.—Often a sequela of exanthematous diseases, and frequently a result of other irritating causes, in those inheriting bad constitutions. The discharge is oftentimes very offensive, and the disease is always a dangerous one. Often it causes necrosis of bone, and may go on to form fistulous openings through the mastoid bone, or even into the cranial cavity; and hearing is always more or less impaired, and that permanently. Treatment.—While we correct any constitutional vice, and also as a rule administer tonics, our therapeutical reliance is mainly placed on the local use of some of the Astringents; Alum is the best, then come Sulphate of Zinc, Sulphate of Copper, and Nitrate of Silver. When in the course of this disease we have deep seated, persistent pain, we may infer that the mastoid cells are invaded, and be called on to trephine the mastoid process. In purulent disease of the middle ear I must strongly insist on the necessity, that the physician should apply the local treatment personally; because no amount of instruction will enable the patient himself, or his friends, thoroughly to cleanse out the ear and apply the remedy. Also, after perfectly cleansing the ear by warm water, he must keep the Eustachian tube open; thus he can blow through the medicated solution after its introduction into the ear, and thus secure its application to every point of diseased membrane—on which contact the efficacy of our treatment *alone* depends. Occasionally also in this trouble, after the discharge is stopped, we find that artificial ear drums decidedly improve the hearing. If we find quite a large aperture in the ear drum, we must not despair; as in children, and especially in cases of recent date, even large sized apertures are frequently filled up by a cicatricial tissue; and the hearing eventually becomes greatly improved.

Inflammation of the Eustachian Tube.—This is often the prominent trouble among children. We must never, however, allow these tubes to remain closed and impervious to air, since as a result by rarefaction, or absorption, of the air in the middle ear, we allow the Membrana Tympani to become deflected inwards, and deafness more or less to supervene. We treat this disease much the same as we would naso-pharyngeal catarrh, or disease of the middle ear, and usually with marked benefit. In addition, however, to other treatment, as often as every second day, we inflate the tube by the catheter or Politzer's bag.

Tinnitus Aurium.—This symptom is of varied etiology, of the most frequent, persistent, least understood, and least amenable to treatment, of any of the features common to diseases of the Ear.

3d. Diseases of the Internal Ear.—Dr. Kramer of Berlin declares that whereas 30 years ago he diagnosed 50 per cent of all diseases of the ear to be those located in the Internal Ear (i. e. nervous disorders of hearing) he is now persuaded that we meet with this class of troubles in only four cases in a thousand. Nervous deafness is indeed rare, and as the occulist has his amaurosis as a refuge, so the Aurist, when through ignorance or when by careful examination he can discover no change in the ear sometimes hides his lack of knowledge by calling the case nervous deafness.

In conclusion, allow me to read a few selections from the last Journal of the American Otological Society:

1st. Account of an inflammation of the external Meatus, caused by the presence therein of the Aspergillus Glaucus—by Dr. John Green.

2d. Description of a new form of Eustachian Catheter—by Dr. Noyes.

3d. An article in relation to inflammation of the Mastoid process, occurring in the course of chronic disease of the Ear—by Dr. Noyes.

Dr. Rider exhibited various instruments for examining and operating upon the ear. He recommended the use of diffuse sunlight, collected by means of a concave mirror, and of Wilde's Silver Speculum.

Society adjourned.

CHARLES S. STARR.

